# Tuberculosis Notes

**Published:** 1921-01-29

**Authors:** 


### TUBERCULOSIS NOTES.

### Yorkshire Tuberculosis Patients—and Fees.

A correspondent of the *Yorkshire Post* has lately drawn attention to certain points in the current West Riding Anti-Tuberculosis Campaign—viz., the long waiting-list of sanatorium patients, the absence of bonus to tuberculosis officers. The waiting-list is given as rather more than the present number under treatment—454 against 444, to be exact. These figures include, as regards waiting-list, 92, and as regards in-patients 155, who are ex-Service men. Happily the number of these awaiting treatment is much lower than was the case two years ago. The County Council is certainly not accused of negligence. In October of last year it decided to provide 300 extra beds, and in July last a resolution was adopted empowering the Public Health and Housing Committee to acquire an estate at a cost not exceeding £20,000 for the treatment of women and children. The correspondent stated that the estate had not yet been acquired, but we believe that this has since been done. Further, 200 patients are accommodated at Middleton Sanatorium, Ilkley. Here additional huts are being put up for fifty patients, and the Ministry of Health are providing a training centre for another fifty. A women's sanatorium has also been enlarged, and beds for surgical tuberculosis leased at Alton and Leasowe. They would like more, but imply that the present demand is greater than the supply. The question of bonus advance for the tuberculosis officers is still under the consideration of the Finance Committee, it is stated, and "it is expected that matters will be adjusted in accordance with the Civil Service award, which gives a bonus on the basic salary to meet increased cost of living."

### Position of Barrasford Sanatorium.

The latest stage in the history of this Northumberland institution for tuberculosis is that a proposal has been made for it to be taken over by a municipality, the Newcastle Corporation. The building was erected, it will be remembered, by the local branch of the National Association for Prevention of Consumption, that is, by private enterprise. The Newcastle Corporation and the Elswick Works soon maintained some beds in the institution, and in spite of some drawbacks in the way of expenses for repairs, the institution began to feel its feet. At this juncture, in the years immediately preceding the war, it was unfortunate that a coalition with the Northumberland county sanitary authority, proposed by the latter body, was not effected. The strain of the war had a very damaging effect, and by the last annual report, a sum of nearly £2,000 had been lost on the year's working. The present-day value put upon the buildings and site is about £50,000, according to the estimate of the Sanitary Committee of the Newcastle Corporation. A complication is introduced by the fact that the sanatorium authorities are entitled in expectancy to a sum of £20,000 under the contingency of the death of an adult, and that the claim of the Corporation to this sum, should they take over the institution, is not wholly free from doubt.

